# Intestinal Microbiota Ecological Response to Oral Administrations of Hydrogen-Rich Water and Lactulose in Female Piglets Fed a *Fusarium* Toxin-Contaminated Diet

**DOI:** 10.3390/toxins10060246

**Published:** 2018-06-16

**Authors:** Weijiang Zheng, Xu Ji, Qing Zhang, Wen Yao

**Affiliations:** 1 Laboratory of Gastrointestinal Microbiology, Jiangsu Key Laboratory of Gastrointestinal Nutrition and Animal Health, College of Animal Science and Technology, Nanjing Agricultural University, Nanjing 210095, China; zhengweijiang@njau.edu.cn (W.Z.); jixuchance@gmail.com (X.J.); zhangqingzee@163.com (Q.Z.); 2 Key Lab of Animal Physiology and Biochemistry, Ministry of Agriculture, Nanjing 210095, China

**Keywords:** intestinal microbiota, hydrogen-rich water, lactulose, *Fusarium* mycotoxins, piglets

## Abstract

The objective of the current experiment was to explore the intestinal microbiota ecological response to oral administrations of hydrogen-rich water (HRW) and lactulose (LAC) in female piglets fed a *Fusarium* mycotoxin-contaminated diet. A total of 24 individually-housed female piglets (Landrace × large × white; initial average body weight, 7.25 ± 1.02 kg) were randomly assigned to receive four treatments (six pigs/treatment): uncontaminated basal diet (negative control, NC), mycotoxin-contaminated diet (MC), MC diet + HRW (MC + HRW), and MC diet + LAC (MC + LAC) for 25 days. Hydrogen levels in the mucosa of different intestine segments were measured at the end of the experiment. Fecal scoring and diarrhea rate were recorded every day during the whole period of the experiment. Short-chain fatty acids (SCFAs) profiles in the digesta of the foregut and hindgut samples were assayed. The populations of selected bacteria and denaturing gradient gel electrophoresis (DGGE) profiles of total bacteria and methanogenic *Archaea* were also evaluated. Results showed that *Fusarium* mycotoxins not only reduced the hydrogen levels in the caecum but also shifted the SCFAs production, and populations and communities of microbiota. HRW treatment increased the hydrogen levels of the stomach and duodenum. HRW and LAC groups also had higher colon and caecum hydrogen levels than the MC group. Both HRW and LAC protected against the mycotoxin-contaminated diet-induced higher diarrhea rate and lower SCFA production in the digesta of the colon and caecum. In addition, the DGGE profile results indicated that HRW and LAC might shift the pathways of hydrogen-utilization bacteria, and change the diversity of intestine microbiota. Moreover, HRW and LAC administrations reversed the mycotoxin-contaminated diet-induced changing of the populations of *Escherichia coli (E. coli)* and *Bifidobacterium* in ileum digesta and hydrogen-utilizing bacteria in colon digesta.

## 1. Introduction

*Fusarium* mycotoxins are secondary metabolites of fungi, produced by several kinds of *Fusarium* species that occur naturally worldwide in cereal grains and animal feed [1]. Among the *Fusarium* mycotoxins, deoxynivalenol (DON) and zearalenone (ZEN) are of special importance as they are formed under predisposing environmental conditions in the field prior to harvest and cannot be completely avoided by strategies [2]. Consumption of *Fusarium* mycotoxins is widely considered as a serious health hazard issue for both animals and human beings, due to their potent toxicity on the gastrointestinal (GI) tract, with symptoms including nausea, vomiting, diarrhea, and oxidative damage [3]. The GI tract is the place where mycotoxin absorption and metabolism occur. Therefore, interactions between the mycotoxins and intestinal microbiota play a major role in the toxicology of mycotoxins [4]. It has been shown that intestinal bacteria are able to bind, transform, degrade, and transfer mycotoxins *in vivo* and *in vitro* [5]. Increasingly attention has been paid on how mycotoxin exposure impacts the intestinal microbiota. Previous studies have shown that the profiles and biodiversity of intestinal microbiota were rapidly and clearly modified in pig exposure to a mycotoxin-contaminated diet [6,7]. Considering the functional effects of microbiota, many methods have been attempted to balance gut microbiota to normalize microbiota in intestinal tracts and keep the host healthy. For example, supplementation of nutritional elements, probiotics, and prebiotics or symbiotic, etc.

Hydrogen gas is one of the metabolites of bacterial fermentation in the gut, which has been proven to act as a novel antioxidant [8]. It has been demonstrated that H_2_ could penetrate cytoplasmic membranes, targets intracellular organelles, and selectively neutralizes cytotoxic reactive oxygen species (ROS) [9]. As a result, hydrogen gas has been applied in many disease models, such as dextran sodium sulfate (DSS)-induced colon inflammatory [10] and intestinal ischemia-reperfusion injuries [11]. A recent report described that 70% of GI microbial species that encoded genetic capacity to metabolize H_2_ [12], indicating that H_2_ levels might affect the gut microbial activity, population, or community. Interestingly, one recent study reported that molecular hydrogen-dissolved alkaline electrolyzed water (AEW) had a strong antimicrobial effect and significantly reduced populations of pathogenic bacteria, such as *Escherichia coli* O157 and *Salmonella* [13]. Xiao et al. also reported that HRW oral gavage resulted in retention of the total abdominal irradiation (TAI)-shifted intestinal bacterial composition in mice [14]. As we know, H_2_ is produced continuously under a normal physiological condition in mammalian animals, primarily during the fermentation of non-digestible carbohydrates by bacteria in the large intestine. Therefore, administration of hydrogen-producing prebiotics might also be a viable method to provide functional H_2_ to animals and humans. Lactulose is a synthetic non-absorbable disaccharide consisting of fructose and galactose [15]. It has been reported that microbial fermentation of lactulose in the hindgut could induce dramatic amounts of endogenous H_2_, providing beneficial effects on liver regeneration [16] and cerebral ischemia-reperfusion injury [17] in rats. In addition, lactulose administration could also lead to the production of short-chain fatty acids (SCFAs) and methane, increasing the diversity and creating a distinct microbiota community in piglets [18,19].

Although HRW and LAC have shown the modifications of microbiota, the effects of HRW or LAC on mycotoxin-induced microbiota imbalance in piglets have not ever been studied. Here, the rationale underlying this study is that HRW or LAC might maintain the intestinal microbiota communities and bacteria populations, thus affecting SCFAs production and eventually improving the health status of female weaning piglets. Therefore, the impacts of HRW and LAC on fecal scoring, diarrhea rate, SCFAs production, intestinal microbiota communities, and populations in piglets fed a *Fusarium* mycotoxin-contaminated diet were explored in this study.

## 2. Results

### 2.1. Hydrogen Levels in Intestinal Segments

**Figure 1:**
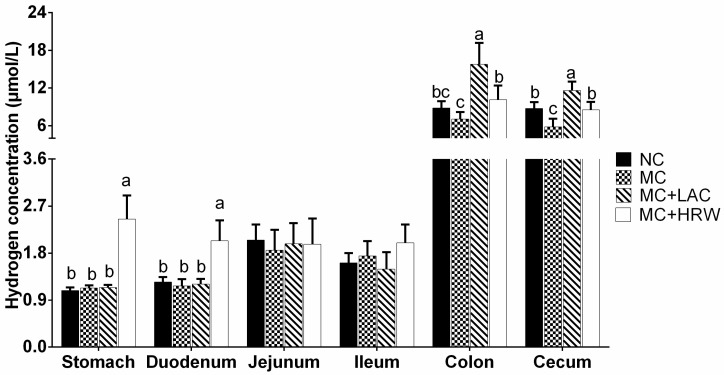
Effects of hydrogen-rich water and lactulose on hydrogen concentrations in different intestinal segments of female piglets fed a *Fusarium* mycotoxin-contaminated diet. Each column represents the mean hydrogen levels with five independent replications, mean ± SD. Letters a–c above the bars indicate statistical significance (*p* < 0.05) among the four treatments. NC (negative control), basal diet; MC, *Fusarium* mycotoxin-contaminated diet; MC + LAC, MC diet + lactulose treatment; and MC + HRW, MC diet + hydrogen-rich water treatment.

At the end of the experiment, that is, day 25 of the experiment, hydrogen concentrations in the mucosa of stomach, duodenum, jejunum, ileum, colon, and caecum are presented in Figure 1. Results showed that stomach and duodenum in the MC + HRW group had the highest hydrogen levels among the four groups (*p* < 0.05), while no difference was found among the other three groups (*p* > 0.05). No difference was found in the jejunum and ileum hydrogen concentrations among the four groups (*p* > 0.05). *Fusarium* mycotoxin-contaminated diet was found to reduce the hydrogen levels in the caecum (*p* < 0.05), but have no impact on the hydrogen levels in the colon (*p* > 0.05). Interestingly, both LAC and HRW treatments significantly increased the hydrogen levels either in the colon or caecum when they compared with the MC group (*p* < 0.05). Furthermore, the MC + LAC group had higher hydrogen levels than the MC + HRW group in the colon and caecum samples (*p* < 0.05).

### 2.2. Fecal Scoring and Diarrhea Rate

The effects of HRW and LAC on fecal scoring and diarrhea rate in piglets fed a *Fusarium* mycotoxin-contaminated diet are shown in Table 1. From days 0 to 7 and days 7 to 14, the *Fusarium* mycotoxin diet was found to significantly increase the fecal score (*p* < 0.05), while HRW and LAC treatments lowered the fecal score from days 0 to 7 and days 7 to 14, respectively (*p* < 0.05). From days 14 to 21, days 21 to 25, and days 0 to 25, no significant difference was found on fecal scoring among the four groups (*p* > 0.05). No difference was found on the diarrhea rate from different time periods except from days 0 to 7 (*p* > 0.05). Compared with the NC group, the diarrhea rate was significantly increased in both MC and MC + LAC from days 0 to 7 (*p* < 0.05), while HRW treatment decreased the diarrhea rate compared with the MC group (*p* < 0.05). Additionally, there was no difference between the MC and MC + LAC groups (*p* > 0.05). 

### 2.3. Short-Chain Fatty Acids (SCFAs) Levels in the Digesta of Jejumun, Ileum, Colon, and Caecum

There was no difference (*p* > 0.05) in the pH value of different intestine segments among the four groups (Table S1). The concentrations of SCFAs levels in different intestinal segments among the treatments are shown in Table 2. In the jejunum and ileum, the levels of acetate, propionate, butyrate, and total SCFAs were found to be no different among the four groups (*p* > 0.05). In the colon, the acetate levels were not influenced by treatments (*p* > 0.05), but the propionate, butyrate, valeric acid, and total SCFAs concentrations in the MC group were lower than the NC group (*p* < 0.05). Compared with the MC group, HRW treatment had higher levels of butyrate (*p* < 0.05) and no effects on colon propionate, valeric acid, and total SCFAs status (*p* > 0.05). However, LAC administration significantly increased the levels of propionate, butyrate, valeric acid, and total SCFAs (*p* < 0.05) when they compared with the MC group. In the caecum, the *Fusarium* mycotoxin-contaminated diet significantly reduced the concentrations of acetate, propionate, butyrate, valeric acid, and total SCFAs (*p* < 0.05). Compared with the MC group, the LAC group had higher concentrations of acetate, propionate, butyrate, and total SCFAs (*p* < 0.05), and the HRW group had higher levels of acetate, butyrate, and total SCFAs (*p* < 0.05).

### 2.4. Microbiota Communities of Total Bacteria and Methanogenic *Archaea* in the Digesta of Intestinal Segments

**Figure 2:**
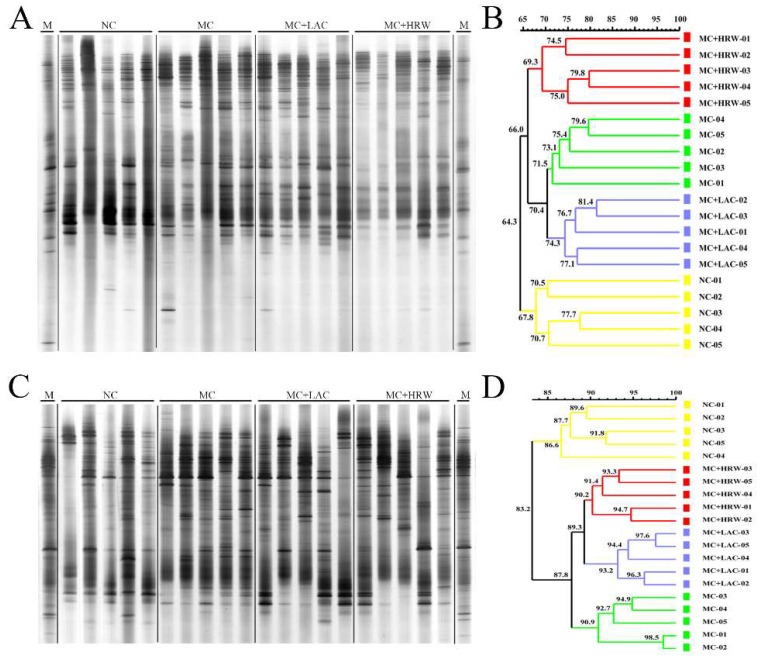
Effects of hydrogen-rich water and lactulose on jejunum and ileum digesta PCR-DGGE profiles of V6–V8 amplicons and similarities index of female piglets fed a *Fusarium* mycotoxin-contaminated diet. (**A**) DGGE profile of bacteria community in the jejunum digesta of four groups; (**B**) Similarity index of bacteria DGGE profile obtained from jejunum digesta of four groups; (**C**) DGGE profile of bacteria community in the ileum digesta of four groups; (**D**) Similarity index of bacteria DGGE profile obtained from ileum digesta of four groups. NC (negative control), basal diet; MC, *Fusarium* mycotoxin-contaminated diet; MC + LAC, MC diet + lactulose treatment; and MC + HRW, MC diet + hydrogen-rich water treatment.

**Figure 3:**
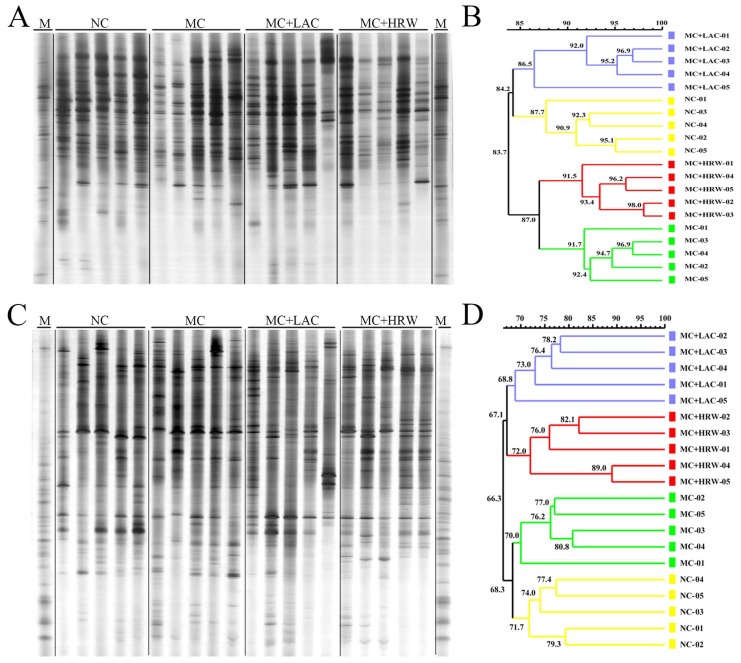
Effects of hydrogen-rich water and lactulose on colon and caecum digesta PCR-DGGE profiles of V6–V8 amplicons and similarities index of female piglets fed a *Fusarium* mycotoxin-contaminated diet. (**A**) DGGE profile of bacteria community in the colon digesta of four groups; (**B**) Similarity index of bacteria DGGE profile obtained from colon digesta of four groups; (**C**) DGGE profile of bacteria community in the caecum digesta of four groups; (**D**) Similarity index of bacteria DGGE profile obtained from caecum digesta of four groups. NC (negative control), basal diet; MC, *Fusarium* mycotoxins-contaminations diet; MC + LAC, MC diet + lactulose treatment; and MC + HRW, MC diet + hydrogen-rich water treatment.

Representative DGGE analysis of total bacteria in foregut (jejunum and ileum) and hindgut (colon and caecum) are shown in Figure 2A,C and Figure 3A,C, respectively. DGGE profiles of PCR products in the V6–V8 regions of the 16S rRNA gene from the four groups revealed some difference among different treatments at the foregut and hindgut. It is shown that jejunum (Figure 2A), ileum (Figure 2C), and colon (Figure 3A) amplicons migrating to the top of the gel were predominant in the samples of all groups, and caecum amplicons (Figure 3C) migrating to the middle of the gel were predominant in the samples of all treatment.

Cluster analysis revealed that the overall similarity of total bacteria DGGE patterns in the jejunum, ileum, colon, and caecum were 64.3%, 83.2%, 83.7%, and 66.3%, respectively (Figure 2B,D, Figure 3B,D, respectively). In the jejunum and ileum, the NC group samples formed in one cluster with a similarity of 67.8% and 86.6%, while MC, MC + LAC, and MC + HRW groups were in another cluster with a similarity of 66.0% and 87.8%, respectively. In the colon, NC and MC + HRW groups formed in one cluster with 84.2% similarity, and MC formed another cluster with the MC + LAC group. However, the cluster pattern in the caecum was quite different, in which NC and MC groups were found in one coherent cluster with 68.3% similarity, and MC + LAC with MC + HRW groups formed another cluster with 67.1% similarity.

**Figure 4:**
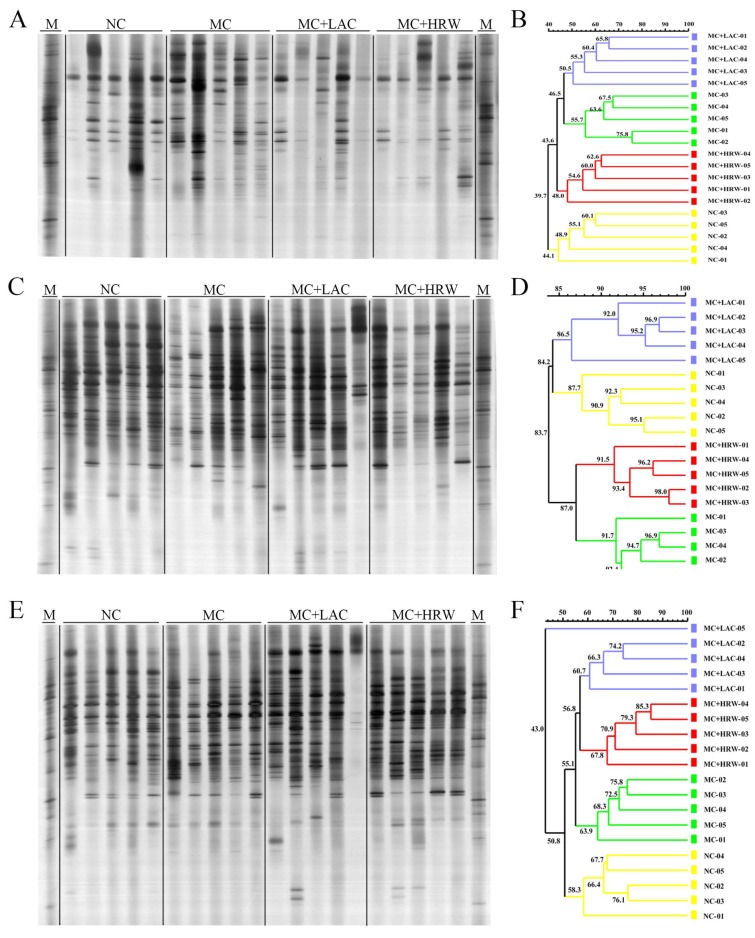
Effects of hydrogen-rich water and lactulose on the ileum, colon and caecum digesta PCR-DGGE profiles of methanogenic *Archaea* and the similarities index of female piglets fed a *Fusarium* mycotoxin-contaminated diet. (**A**) DGGE profile of methanogenic *Archaea* community in the ileum digesta of four groups; (**B**) Similarity index of methanogenic *Archaea* DGGE profile obtained from ileum digesta of four groups; (**C**) DGGE profile of methanogenic *Archaea* community in the colon digesta of four groups; (**D**) Similarity index of methanogenic *Archaea* DGGE profile obtained from colon digesta of four groups; (**E**) DGGE profile of methanogenic *Archaea* community in the caecum digesta of four groups; (**F**) Similarity index of methanogenic *Archaea* DGGE profile obtained from caecum digesta of four groups. NC (negative control), basal diet; MC, *Fusarium* mycotoxins-contaminations diet; MC + LAC, MC diet + lactulose treatment; and MC + HRW, MC diet + hydrogen-rich water treatment.

For the methanogenic *Archaea* compositions, the ileum (Figure 4A), colon (Figure 4C), and caecum (Figure 4E) amplicons migrating to the top of gels were predominant in the samples of all groups. DGGE cluster analysis showed that the overall similarity in the ileum, colon, and caecum were 39.7%, 83.7%, and 43.0%, respectively. In the ileum (Figure 4B), the NC group formed one cluster with a similarity of 44.1%, while MC, MC + LAC, and MC + HRW groups formed another cluster with a similarity of 43.6%. In the colon (Figure 4D), the methanogenic *Archaea* pattern is different than the ileum, in which NC and MC + LAC treatments formed one cluster with 84.2% similarity, while MC and MC + HRW groups formed another cluster with 87.2% similarity. In the caecum (Figure 4F), the NC group formed one cluster with 58.3% similarity. Except for one sample from the MC + LAC group, the MC, MC + HRW, and MC + LAC groups formed another cluster with 55.1% similarity.

The band number and Shannon diversity index of total bacteria and methanogenic *Archaea* were also analyzed and are shown in Table 3. For total bacteria, the band numbers and Shannon diversity in the jejunum were not affected by treatments (*p* > 0.05). In the ileum, *Fusarium* mycotoxins did not affect the band numbers (*p* > 0.05), but MC + LAC group had more band numbers than the MC group (*p* < 0.05). The Shannon diversity was found to be significantly increased by *Fusarium* mycotoxin treatment, while neither LAC nor HRW influenced the Shannon diversity (*p* > 0.05). In the colon, Shannon diversity found no differences among the treatments (*p* > 0.05). MC and MC + LAC groups were found to have higher band numbers than the NC and MC + HRW groups (*p* < 0.05), and no difference was found neither between the MC and MC + LAC groups nor between the NC and MC + HRW groups (*p* > 0.05). In the caecum, Shannon diversity did not find any differences among the four groups. Band numbers in the MC, MC + LAC, and MC + HRW groups were significantly higher than the NC group (*p* > 0.05), while no difference was found among the MC, MC + LAC, and MC + HRW groups (*p* < 0.05).

For methanogenic *Archaea*, the band number from the ileum, colon, and caecum digesta in the MC group were significantly higher than the NC group (*p* < 0.05), and both LAC and HRW were found to reduce those band number compared with MC group (*p* < 0.05). The Shannon diversity of ileum digesta in the MC group was significantly higher than the NC group (*p* < 0.05), and MC + LAC and MC + HRW were found to have lower Shannon diversity than the MC group (*p* < 0.05). The Shannon diversity of colon and caecum digesta showed no significant differences among the four groups (*p* > 0.05).

### 2.5. Populations of Selected Bacteria in the Digesta of Different Intestinal Segments

Table 4 shows the populations of selected microbiota in the digesta of the jejunum, ileum, colon, and caecum in piglets fed a *Fusarium* mycotoxin-contaminated diet. In the jejunum and caecum, no difference was found on the abundance of selected bacteria among the four treatments (*p* > 0.05). In the ileum, the populations of total bacteria, *Lactobacillus*, and *Enterococcus* were not impacted by treatments, but the abundance of *E. coli* in the MC group was higher than NC, MC + LAC, and MC + HRW groups (*p* < 0.05). The MC group also had a lower (*p* < 0.05) *Bifidobacterium* abundance than the NC, MC + LAC, and MC + HRW groups, while no difference was found among the latter three groups (*p* > 0.05). In the colon, the populations of total bacteria and acetogenic bacteria showed no significant difference among the four groups (*p* > 0.05). However, the abundance of methanogenic *Archaea* and sulfate-reducing bacteria (SRB) was lower in the MC group than the NC group (*p* < 0.05). In addition, MC + HRW and MC + LAC groups were found to have higher populations of these two selected species than the MC group (*p* < 0.05).

## 3. Discussion

The gut microbiota ecological system represents an abundant community of organisms, including bacteria, archaea, fungi, and viruses, that colonized within the GI tract [20]. The complex and large diverse communities of the microbiota play important roles in nutrition decomposition and transformation, immunity intrusion, and gut villous structure development through a symbiotic relationship with the host [20]. Diet is a major factor that influences the makeup and activity of colonized microbes in the intestinal gut, and we had hypothesized that *Fusarium* mycotoxins could induce compositions and/or metabolites changes in the gut microbiota, and eventually affect the health of female piglets. In addition, oral HRW or lactulose administrations may partly spare the harmful activity of *Fusarium* mycotoxins on the intestinal microbiota ecosystem of female weaning piglets. These results partly support our hypothesis.

### 3.1. Hydrogen Levels in Intestinal Segments

One major function of microbiota in the gut lumen is the fermentation of indigestible polysaccharides, dietary fiber, and resistant starch, which generates large quantities of H_2_ [21]. In this study, our data showed that administration of HRW increased concentrations of H_2_ in the stomach, duodenum, colon, and caecum. However, lactulose treatment was found to have no impact on the H_2_ levels in the foregut, but significantly increased the concentrations of H_2_ in the hindgut (colon and caecum). The communities, species, and population of microbiota in the hindgut were more complex than the foregut and might contribute to this difference [22]. In fact, these observed H_2_ concentrations in the intestine were similar to those in a study reported by Liu et al. [23], who recently developed a conventional gas chromatography method to measure the hydrogen levels in the tissue following administration of HRW, hydrogen-rich saline and inhalation of hydrogen gas at different time points. Their data indicated that after 30 min of administration, hydrogen gas could also be detected at around 10–20 ppb/g intestine tissue. Moreover, oral administration of HRW had the highest Cmax compared with other methods [23]. Watanabe et al. found that inhalation of hydrogen gas resulted in slower elevation than that achieved with intraperitoneal administration [24], indicating that different administration ways of providing hydrogen may result in different hydrogen distributions in blood and tissues. On the other hand, previous studies also indicated that oral administration of hydrogen-rich water/saline had very short lasting time [25]. Thus, the protective effects of HRW in this study may not yet be fully exerted. Therefore, the search for an ideal hydrogen-producing prebiotic may be an interesting method to solve this concern.

Lactulose is a semisynthetic disaccharide and cannot be metabolized nor absorbed in the intestine [15]. Like other polysaccharides, lactulose is fermented in the gastrointestinal gut by bacteria, producing SCFAs. Hydrogen gas is one of its metabolites, which may act as an effective ideal agent [26]. A higher breath H_2_ can be detected after oral administration of lactulose [17]. After lactulose administration, the exhaled breath H_2_ concentration was found to be increased after 15 min and reaching a peak at 45 min in mice; meanwhile, lactulose with antibiotic treatment did not find an improvement of breath hydrogen levels [27]. On one hand, a proportion of hydrogen gas may pass through the gut mucosa wall into the blood and be transported to other organs. On the other hand, hydrogen also can be metabolized by intestinal microbiota. Yu et al. also demonstrated that lactulose significantly increased the accumulated hydrogen levels, and antibiotic administration induced a smaller amount of H_2_ in rats [16]. These results indicate that bacterial fermentation may play a curial role in the beneficial effects of lactulose in animals. Considering the features of LAC, it is broadly in line with our expectations that lactulose administration had a continual improvement in the hydrogen concentrations in the mucosa of the hindgut (colon and caecum). Although higher hydrogen levels were both observed in HRW (stomach and duodenum) and LAC (colon and caecum) treatments compared with MC group, it is not representative of all H_2_ production of HRW or LAC. Thus, more research needs to be carried out to demonstrate the dynamic changes of hydrogen gas in the intestine after receiving hydrogen-rich water or lactulose in piglets.

### 3.2. Fecal Scoring and Diarrhea Rate

Disruptions of the microbiota ecological system under abnormal conditions is recognized as a major risk factor for various diseases, such as diarrhea and inflammatory bowel disease. In the swine industry, weaning piglets face enormous stress, leading to perturbations in gut microbiota, and host physiological and mucosal immune function, such as microbiological, environmental, and dietary factors. The transition from liquid to solid feeding is expected to result in decreased feed intake and daily weight gain, and increased diarrhea incidence [28]. Corn and soybean meal represent over 50% of the total dietary ingredients, which are often contaminated with mycotoxins [29]. Thus, post-weaning piglets are always associated with diarrhea or loose feces [30]. Consumption of DON- and ZEN-contaminated feeds will induce intoxication symptoms, including vomiting, abdominal distress, malaise, diarrhea emesis, and even shock or death [3]. In the present study, lower feed intake and weight gain [31], fever, and severe diarrhea were observed in piglets fed a *Fusarium* mycotoxin-contaminated diet. A lower fecal score has been considered a good indicator of gut health [32]. Feces scoring was decreased in piglets fed a *Fusarium* mycotoxin-contaminated diet in the critical period of days 0 to 7 and days 7 to 14 after weaning. In addition, the MC group had higher diarrhea rates than the NC group in the first week. These results are consistent with previous studies in which DON has the capacity to increase intestinal permeability, and cause microbiota dysfunctions which promote intestinal disorders [3,33]. It is also reported that DON ingestion was associated with the reduction of mRNA expression of Na^+^-dependent glucose transport sodium-dependent glucose cotransporter 1 (SGLT1) [34]. Since SGLT1 is responsible for water reabsorption, inhibition of SGLT1 could also cause diarrhea, which might be the underlying cause of diarrhea in animals exposed to mycotoxins [34].

A number of studies have demonstrated that prebiotics may selectively increase the population and/or activity of beneficial bacteria [35], decreasing the incidence of diarrhea. Lactulose is always used in the treatment of constipation and hepatic encephalopathy in humans [15]. However, at low doses, lactulose acts as a prebiotic that improves growth performance and intestinal morphology in pigs [36]. A previously study on piglets showed that 1% lactulose supplementation had higher growth performance [37]. Additionally, our previous study also suggested that lactulose could improve anti-oxidant capacity of piglets fed a mycotoxin-contaminated diet [31]. Although no studies explored the effects of HRW on diarrhea, a protective effect of HRW was found on DSS-induced inflammatory bowel disease (IBD) in rats, and intestinal villi damage in mice [10]. The exact mechanisms underlying the protective effects of HRW and LAC on diarrhea is still unclear, but might be attributed to the ability of both HRW and lactulose could remedy mycotoxin-induced intestine damage through the anti-oxidant and anti-inflammatory properties. Therefore, it is not surprising that piglets orally administered HRW or lactulose had a lower fecal score and incidence of diarrhea than the MC group.

### 3.3. SCFAs Levels in the Digesta of Jejumun, Ileum, Colon, and Caecum

Shifting of the microbiota composition and quality of intestinal microbiota, followed by the alternation in SCFAs levels and other metabolites, may have a role in ensuring animal health and disease [38]. Here, we found a significant inhibitory effect of *Fusarium* mycotoxins on the production of SCFAs in the colon and caecum, which have not been previously reported. Ingestion of DON contaminated diet was found to significantly reduce feed intake and increase diarrhea [3], which would increase the water levels and reduce the dry matter content in the digesta. In fact, compared with the NC group, a lower caecum relative weight was observed in the MC group (Figure S1). Therefore, it is conceivable that *Fusarium* mycotoxins (DON and ZEN) may have modified the microbiota fermentation in the hindgut due to changes in the colon and caecum nutrient flow and water content. In addition, the hydrogen levels in the hindgut were also reduced by *Fusarium* mycotoxin treatment, which may support the changes of SCFAs production.

As a prebiotic, lactulose is not broken down by mammalian intestine enzymes, but can be metabolized by gut microbiota to SCFAs. A previous study in pigs revealed that the inclusion of lactulose in the diet increased the SCFAs concentration in the large intestine [39]. Increased SCFAs concentrations were also observed in broiler chickens provided with lactulose supplementation [40]. However, Martin-Pelaez et al. found that 1% dietary lactulose supplementation did not affect caecum butyric acid concentration in piglets that were orally challenged with *Salmonella* [41]. Recently, it has been shown that the dietary inclusion of 1% lactulose did not change the branched-chain fatty acids (BCFA) levels in piglets [37]. Our data suggests that lactulose modified the hindgut microbiota fermentation, resulting in higher SCFAs production. So far, the relationship of hydrogen-rich water on short-chain fatty acid production in vivo and in vitro are unknown*.* Further studies are warranted to explore the effects of HRW and lactulose on bacteria fermentation in piglets.

### 3.4. Microbiota Communities and Populations

It is well known that the dynamic complex intestinal microbial ecosystem plays key roles in maintaining host nutritional, physiological, and immunological functions [38]. Thus, impairment of microbiota balance could have many adverse effects on the health of the host. Mycotoxins not only undergo microbial metabolism in the gastrointestinal tract, but may also affect the communities due to some toxins exhibiting antimicrobial properties [34,42]. *In vitro* studies showed that DON did not influence the growth of *Staphylococcus aureus*, *E. coli*, and *Yersinia enterocolitica* [43]. It has been reported that consumption of feed contaminated with a moderate level of DON had a slight effect on cultivable bacteria in pig intestines, and the composition of intestinal microbiota was also observed in DON-exposed animals [44]. Feeding pigs with T-2 toxin led to a substantial increase of aerobic bacterial counts in the intestine [45]. Similarly, chronic exposure of pigs to low doses of DON caused an increase in the number of intestinal aerobic bacteria and modified the dynamics of intestinal bacteria communities [6]. Piotrowska et al. also reported the effect of exposure of pigs to the *Fusarium* mycotoxins ZEN and DON, administered together and separately, on the colon microbiota [7]. After 42 days of experiment, their data found that ZEN alone, and together with DON, had an adverse effect on mesophilic aerobic bacteria. The concentration of *C. perfringens*, *E. coli*, and other bacteria in the family *Enterobacteriaceae* was significantly reduced by ZEN alone, and together with DON. The functional biodiversity of microorganisms was also affected by mycotoxins, which Shannon’s diversity index was higher [7]. In our study, cluster results of DGGE profiles obtained from the jejunum, ileum, colon, and caecum showed that the mycotoxin-contaminated diet drastically affected the communities, diversity, and population of gastrointestinal microbiota (especially in the ileum and colon). Moreover, our data also indicated that hydrogen-utilizing bacteria (methanogenic *Archaea* and SRB) were also involved in the mycotoxincosis-induced intestinal microbiota dysbiosis.

Previous *in vivo* studies demonstrated positive protective effects of lactulose on colon fermentation [46]. Lactulose was reported to increase the number of *Bifidobacteria* and *Lactobacilli* while reducing the numbers of *Clostridium* spp., *Salmonella* spp., or *E. coli* in the pig gastrointestinal tract [36]. Guerra-Ordaz et al. demonstrated that lactulose significantly improved the performance and colonic microbial activity of weaning piglets [37]. In a previous study, lactulose was included in the diet at 1%, 0.2%, 0.4%, 0.6%, or 0.8% at the expense of corn and/or soybean meal. A significant quadratic response in the *Lactobacillus* count was observed at 42 days on increasing the level of lactulose [40]. However, the effects of HRW on intestinal microbiota in piglets are quite limited. In a mouse study, 16S rRNA gene sequencing analysis was introduced to explore the effects of AEW on the microbial composition of C57BL/6N mice. After four weeks of treatment, the relative abundance of 20 taxa differed significantly in AEW-administered mice [47]. Xiao et al. also reported that hydrogen-water oral gavage resulted in retention of TAI–shifted intestinal bacterial composition in mice by high-throughput sequencing [14], which is consistent with our results. In the present, our data showed that both lactulose supplemental could influence the communities and diversity of bacteria and methanogenic *Archaea* in different segments of piglets fed a mycotoxin-contaminated diet. In addition, the shifts of the abundance of *Bifidobacterium* and *E. coli* in the ileum digesta, and hydrogen-utilizing bacteria in the colon were also attenuated by both HRW and LAC treatments.

## 4. Conclusions

In this study, we found that *Fusarium* mycotoxin significantly affected the metabolism, activities, and communities of gut microbiota, and eventually caused a higher diarrhea rate in female piglets. Most importantly, both hydrogen-rich water and lactulose have shown protective effects on the imbalance of intestinal microbiota, reducing the SCFAs production and the higher diarrhea rate induced by *Fusarium* mycotoxin-contaminated diet, partly through affecting the communities/populations of microbiota and the evaluation of hydrogen gas. These results partly support our original hypothesis.

## 5. Materials and Methods

### 5.1. Preparation of Fusarium Mycotoxin-Contaminated Maize

The *Fusarium graminearum* strain 2021 was cultured and conidia were prepared as previously described [48]. Commercial maize was soaked in tap water for 72 h, and autoclaved at 121 °C for 30 min. Then, the maize was incubated in plastic storage boxes with 1 × 10^6^ conidia/kg for 30 days (15–25 °C and 50–85% humidity). The cool autoclaved maize without conidia was used as the control maize. Finally, uncontaminated and *Fusarium graminearum*-contaminated maize were dried in an oven at 70 for 24 h, respectively.

### 5.2. Experimental Diets and Mycotoxins Analyusis

*Fusarium* mycotoxin-contaminated maize and uncontaminated control maize were used at 44.5% at the expense of normal maize for the manufacturing of two experimental diets, respectively. The experimental diets (negative control (NC) and mycotoxin-contaminated diet (MC), respectively) were formulated according to the recommendation of the nutrient requirement of swine by the National Research Council [49] and based on a previous study [50] with minor modifications to the vitamin and mineral premix.

No antibiotic, hormone, and preservatives were added to the diets. Table S2 shows the ingredients of the two experimental diets used in this study. The analysis of *Fusarium* mycotoxin levels in the two experimental diets were described in our previous study [31]. The NC diet contained 221.10 μg/kg DON, 12.12 μg/kg 3-acetyl DON, 32.95 μg/kg 15-acetyl DON, and 266.26 μg/kg total DON. The MC diet contained 825.46 μg/kg DON, 212.79 μg/kg 3-acetyl DON, 59.45 μg/kg 15-acetyl DON, 1097.99 μg/kg total DON, and 501.56 μg/kg ZEN, which were each significantly higher (*p* < 0.05) than in the NC diet. In the current study, the levels of DON in the contaminated diet were expected in natural conditions, which is similar with a previous Chinese report showing the mean DON levels were 753.1–1194.0 μg/kg and the maximum levels reached to 4279.3 μg/kg in complete pig feed between 2016 and 2017 [51].

### 5.3. Animals

In the swine industry, weaning piglets face enormous stress, which leads to perturbations in gut microbiota, host physiological, and mucosal immune function, such as microbiological, environmental, and dietary factors [28]. In addition, corn and soybean meal represent over 50% of the total dietary ingredients, which is often contaminated with mycotoxins [29], which will aggravate the post-weaning stress and induce higher economic losses. Therefore, piglets are a good model to explore the effects of *Fusarium* mycotoxins on microbiota. Furthermore, finding a way to minimize the side effects of mycotoxins on piglets should be valuable for swine production.

Therefore, a total of 24 clinically-healthy female weaning piglets (Landrace × large × white; initial average body weight, 7.25 ± 1.02 kg) from six litters (four pigs/little) were individually housed in pens (1.2 by 2.0 m) with one feeder and one nipple drinker. The piglets had ab libitum access to feed and water. This protocol was approved by the Committee of Animal Research Institute (Certification No. SYXK(Su)2011-0036, 11 August 2015), Nanjing Agricultural University, China.

### 5.4. Experimental Design and Sampling

After a six day adaption period, the animals were randomly assigned to four treatment groups (NC, MC, MC + HRW and MC + LAC, respectively), with six piglets in each group. The piglets were fed their corresponding experimental diets for 25 days. The piglets in the NC group were fed an uncontaminated control diet, while piglets in the MC, MC + HRW, and MC + LAC groups were fed *Fusarium* mycotoxin-contaminated diets.

Piglets in each group received oral administration with their corresponding treatment twice a day (1000 and 1400 h) through a 6 × 200 mm nasogastric tube (Jiangsu Huatai Medical Devices Company, Yangzhou, China). The hydrogen-free water was orally administered at 10 mL/kg body weight (BW) to piglets in both NC and MC groups. While piglets in the MC + HRW group received 10 mL/kg BW HRW (Beijing Hydrovita Biotechnology Company, Beijing, China). The HRW was produced by dissolving high-pressure hydrogen gas into pure water, and kept in 300 mL aluminum pouches at room temperature. At least 0.6 mM levels of hydrogen were detected in the HRW, and they were administered to piglets within 15 min after opening. Lactulose (4-*O*-β-d-galactosyl-d-fructose; formula, C_12_H_22_O_11_; CAS number, 4618-18-2) was used in this study. A dose of 500 mg/kg BW of LAC oral solution (Abbott Healthcare Products, Weesp, The Netherlands) was dissolved in 10 mL/kg BW of hydrogen-free water, and administrated to piglets in the MC + LAC group.

The amounts of LAC and HRW were dependent on the body weight and updated weekly. One piglet was removed from the MC, MC + LAC, and MC + HRW groups fed with a *Fusarium* mycotoxin diet due to poor health condition. Therefore, five independent replicates from each group were used in this study. On day 25, 30 min after administration of different treatments, piglets were euthanized by an intramuscular injection of sodium pentobarbital (40 mg/kg BW). The whole cecum and colon were obtained and weighted. Digesta samples from jejunum, ileum, colon, and caecum were collected and stored at −70 °C for further analysis.

### 5.5. Feces Scoring

Piglets were closely observed daily for clinical signs of diarrhea and a scoring system was applied to indicate the presence and severity of diarrhea as previously described [52]. Feces scoring began on day 0 on the experimental diets and continued until day 25. Scores were given daily watch and the average fecal score value per piglets was given. The following feces scoring system was used: 1 = hard feces, 2 = slightly soft feces, 3 = soft, partially formed feces, 4 = loose, semiliquid feces, and 5 = watery, mucous-like feces.

### 5.6. Hydrogen Gas Measurement in Different Intestine Segments

Hydrogen levels in the mucosa samples from different intestine segments were analyzed using a hydrogen sensor (Unisense, Aarhus, Denmark) as previously described [53]. Briefly, piglets were euthanized with sodium pentobarbital and placed in supine position. Incisions were made in the segments of stomach, duodenum, jejunum, ileum, colon and cecum. The digesta were removed and hydrogen microelectrode (diameter, 50 μm) was penetrated into the mucosa at a depth of 200 μm.

### 5.7. SCFA Detection in the Digesta of Different Intestine Segements

The digesta samples (0.3 g) was weighed and mixed with 0.9 mL of meta-phosphoric acid (25%, *w*/*v*) and crotonic acid (75 mM) solution. The mixture was vortexed and centrifuged at 12,000× *g* for 10 min at 4 °C, and the supernatant was stored at −20 °C until assay. After thawing, the supernatant was centrifuged at 12,000× *g* for 5 min, and the supernatant was used for SCFA detection. The supernatant was detected by using a capillary column gas chromatograph (GC-14A with an FID detector; Shimadzu, Japan; capillary column: 30 m × 0.32 mm × 0.25 μm film thickness) with a H_2_ flame ionization detector and split injection as previously described [54]. The column, injector, and detector temperature were 140 °C, 180 °C, and 180 °C, respectively.

### 5.8. DNA Isolation, PCR Amplification, and DGGE Analysis

Total DNA of the jejunum, ileum, colon, and caecum digesta samples were extracted by a QIAamp^®^ DNA Stool Mini Kit (Qiagen, Germany) according to the manufacturer’s instructions. PCR products of the total bacteria and methanogenic *Archaea* for denaturing gradient gel electrophoresis (DGGE) analysis were amplified with specific primers (Table S3) and separated in denaturing gradient polyacrylamide gels as previously described [55].

### 5.9. Real-Time PCR Assays for Quantification of the Selected Bacteria

The abundance of total bacteria, *Lactobacillus*, *Bifidobacterium*, *Escherichia coli*, *Enterococcus*, methanogenic *Archaea*, sulfate-reducing bacteria, and acetogenic bacteria were quantified by real-time PCR using specific primers (Table S3). Standard curves of each bacterial group were generated using triplicate ten-fold dilutions of known copy numbers of a target gene cloned into a plasmid vector. Real-time PCR was carried out on SetpOnePlusTM Real-Time PCR System (Life Technologies, Carlsbad, CA, USA) by using SYBR Premix Ex Taq (Takara, Dalian, China).

### 5.10. Statistical Analyses

Data from DGGE gels were calculated as previously described [55]. All statistical analyses were performed by one-way ANOVA with SPSS statistical software (version 18.0 for Windows, SPSS Inc., Chicago, IL, USA, 2009). The differences among treatments were considered significant at *p* < 0.05. Differences among treatments were determined using the Tukey-Kramer test.

## Figures and Tables

**Table 1:** Effects of hydrogen-rich water and lactulose on fecal scoring and diarrhea rate of female piglets fed a *Fusarium* mycotoxin-contaminated diet ^1,2^.

Item	NC	MC	MC + LAC	MC + HRW	SEM	*p*-Value
Fecal score						
Days 0–7	2.64 ^c^	3.60 ^a^	3.17 ^b^	2.64 ^c^	0.10	<0.001
Days 7–14	2.84 ^b^	3.74 ^a^	3.12 ^b^	3.02 ^b^	0.12	0.037
Days 14–21	2.78	3.00	2.82	2.95	0.11	0.909
Days 21–25	2.79	2.53	2.82	3.00	0.15	0.758
Days 0–25	2.76	3.30	3.02	2.91	0.09	0.163
Diarrhea rate %						
Days 0–7	5.00 ^b^	30.00 ^a^	25.00 ^a^	5.00 ^b^	3.64	0.008
Days 7–14	5.00	30.00	22.50	15.00	5.00	0.353
Days 14–21	7.50	17.50	15.00	12.50	5.09	0.927
Days 21–25	12.00	0.00	12.00	16.00	5.53	0.788
Days 0–25	6.92	22.31	20.00	12.31	3.65	0.449

^a,b,c^ Values with different letters within the same row are different (*p* < 0.05). ^1^ NC (negative control), basal diet; MC, *Fusarium* mycotoxin-contaminated diet; MC + LAC, MC diet + lactulose treatment; and MC + HRW, MC diet + hydrogen-rich water treatment. ^2^ *n* = 5.

**Table 2:** Effects of hydrogen-rich water and lactulose on short-chain fatty acids (SCFAs) profiles in the jejunum, ileum, colon, and caecum digesta of female piglets fed a *Fusarium* mycotoxin-contaminated diet ^1,2^.

Item	NC	MC	MC + LAC	MC + HRW	SEM	*p*-Value
Jejunum (μmol/g wt digesta)						
Acetate	4.30	3.80	4.48	4.14	0.27	0.819
Propionate	2.08	1.58	1.83	1.99	0.12	0.486
Butyrate	0.94	0.72	0.84	1.00	0.06	0.425
Total SCFAs	7.45	6.10	7.15	7.13	0.43	0.737
Ileum (μmol/g wt digesta)						
Acetate	10.50	10.64	10.58	10.29	0.41	0.993
Propionate	5.09	5.56	5.65	5.16	0.27	0.660
Butyrate	0.49	0.50	0.52	0.54	0.03	0.932
Total SCFAs	16.08	16.69	16.75	15.99	0.66	0.971
Colon (μmol/g wt digesta)						
Acetate	44.28	39.83	49.94	43.78	1.48	0.102
Propionate	19.13 ^a^	11.19 ^b^	20.13 ^a^	14.62 ^ab^	1.17	0.010
Butyrate	8.25 ^a^	4.00 ^b^	7.55 ^a^	7.35 ^a^	0.53	0.008
Valeric acid	4.40 ^a^	1.00 ^c^	2.55 ^b^	0.91 ^c^	0.40	<0.001
Total SCFAs	76.06 ^a^	56.02 ^b^	80.18 ^a^	66.66 ^ab^	2.99	0.008
Caecum (μmol/g wt digesta)						
Acetate	57.29 ^a^	51.47 ^b^	60.45 ^a^	60.91 ^a^	1.14	0.003
Propionate	26.20 ^a^	19.12 ^b^	25.97 ^a^	22.50 ^ab^	0.99	0.020
Butyrate	12.43 ^a^	6.02 ^c^	9.89 ^b^	10.04 ^b^	0.59	<0.001
Valeric acid	9.84 ^a^	2.49 ^b^	2.22 ^b^	3.01 ^b^	0.77	<0.001
Total SCFAs	105.76 ^a^	79.10 ^c^	98.53 ^ab^	96.46 ^b^	2.60	<0.001

^a,b,c^ Values with different letters within the same row are different (*p* < 0.05). ^1^ NC (negative control), basal diet; MC, *Fusarium* mycotoxin-contaminated diet; MC + LAC, MC diet + lactulose treatment; and MC + HRW, MC diet + hydrogen-rich water treatment. ^2^ *n* = 5.

**Table 3:** Effects of hydrogen-rich water and lactulose on the number of DGGE bands and Shannon diversity of the jejunum, ileum, colon, and caecum digesta of female piglets fed a *Fusarium* mycotoxin-contaminated diet ^1,2^.

Target Group	DNA Sample	Item	NC	MC	MC + LAC	MC + HRW	SEM	*p*-Value
Total bacteria	Jejunum	Band number	59.20	60.60	64.60	59.60	1.34	0.498
		Shannon diversity	3.40	3.64	3.68	3.60	0.05	0.278
	Ileum	Band number	34.40 ^c^	37.70 ^bc^	44.40 ^a^	38.40 ^b^	1.00	<0.001
		Shannon diversity	3.00 ^b^	3.27 ^a^	3.49 ^a^	3.34 ^a^	0.06	0.006
	Colon	Band number	40.00 ^b^	50.40 ^a^	52.00 ^a^	40.00 ^b^	1.60	0.001
		Shannon diversity	3.14	3.15	3.14	3.19	0.04	0.948
	Caecum	Band number	61.60 ^b^	72.60 ^a^	72.80 ^a^	71.60 ^a^	4.23	0.036
		Shannon diversity	3.55	3.76	3.75	3.81	0.04	0.070
Methanogens	Ileum	Band number	15.20 ^b^	26.80 ^a^	17.20 ^b^	18.40 ^b^	1.14	<0.001
		Shannon diversity	2.27 ^b^	2.91 ^a^	2.27 ^b^	2.28 ^b^	0.09	0.015
	Colon	Band number	29.80 ^bc^	36.20 ^a^	32.20 ^b^	27.40 ^c^	0.89	<0.001
		Shannon diversity	2.99	3.23	2.87	2.89	0.07	0.208
	Caecum	Band number	31.40 ^b^	41.00 ^a^	32.80 ^b^	34.60 ^b^	1.27	0.023
		Shannon diversity	3.06	3.34	2.92	3.22	0.08	0.234

^a,b,c^ Values with different letters within the same row are different (*p* < 0.05).^1^ NC (negative control), basal diet; MC, *Fusarium* mycotoxin-contaminated diet; MC + LAC, MC diet + lactulose treatment; and MC + HRW, MC diet + hydrogen-rich water treatment. ^2^ *n* = 5.

**Table 4:** Effects of hydrogen-rich water and lactulose on the populations (log copies number/g) of selected bacteria in the digesta of the jejunum, ileum, colon and caecum of female piglets fed a *Fusarium* mycotoxin-contaminated diet ^1,2^.

Sample	Item	NC	MC	MC + LAC	MC + HRW	SEM	*p*-Value
Jejunum	All bacteria	8.75	8.66	8.67	8.20	0.13	0.486
	*Lactobacillus*	7.96	7.43	7.25	7.28	0.19	0.552
	*Bifidobacterium*	4.90	4.76	5.26	5.14	0.08	0.124
	*Escherichia coli*	4.87	5.78	5.53	5.31	0.14	0.127
	*Enterococcus*	3.30	3.45	3.21	3.06	0.11	0.685
Ileum	All bacteria	9.04	9.64	9.43	9.53	0.14	0.221
	*Lactobacillus*	8.85	8.60	8.75	8.86	0.25	0.906
	*Bifidobacterium*	5.07 ^a^	4.23 ^b^	5.26 ^a^	5.13 ^a^	0.13	0.009
	*Escherichia coli*	5.67 ^b^	7.25 ^a^	6.14 ^b^	6.21 ^b^	0.16	0.005
	*Enterococcus*	3.08	3.66	3.39	3.32	0.58	0.488
Colon	All bacteria	11.04	11.09	11.17	10.93	0.07	0.704
	Methanogens	4.49 ^a^	3.47 ^c^	5.52 ^a^	4.58 ^b^	0.20	0.001
	SRB ^3^	4.54 ^a^	3.27 ^b^	4.68 ^a^	4.12 ^a^	0.18	0.011
	Acetogenic bacteria	6.87	6.85	7.17	6.54	0.11	0.297
Caecum	All bacteria	11.57	11.34	11.69	11.60	0.05	0.119
	Methanogens	5.14	5.55	5.44	5.36	0.28	0.969
	SRB ^3^	2.89	3.39	3.02	2.88	0.12	0.470
	Acetogenic bacteria	6.21	6.31	6.32	6.19	0.16	0.992

^a,b,c^ Values with different letters within the same row are different (*p* < 0.05).^1^ NC (negative control), basal diet; MC, *Fusarium* mycotoxin-contaminated diet; MC + LAC, MC diet + lactulose treatment; and MC + HRW, MC diet + hydrogen-rich water treatment. ^2^ *n* = 5. ^3^ SRB = Sulfate-reducing bacteria.

## Supplementary Materials

The following are available online at http://www.mdpi.com/2072-6651/10/6/246/s1, Table S1: Effects of hydrogen-rich water and lactulose on pH values in different intestine segments in piglets fed *Fusarium* mycotoxins contaminated diets, Table S2: Ingredient composition and nutrient contents of control and experimental diets, Table S3: Primers sequences used in this study, Figure S1: Effects of lactulose and hydrogen water on the relative colon and caecum weights of piglets fed *Fusarium* mycotoxins contaminated diet.
