# Supplementary material for: Intestinal Microbiota Ecological Response to Oral Administrations of Hydrogen-Rich Water and Lactulose in Female Piglets Fed a Fusarium Toxin-Contaminated Diet
Source: Toxins (Basel). 2018 Jun 16;10(6):246. doi: 10.3390/toxins10060246 (PMC6024725; doi:10.3390/toxins10060246)
Supplement: Supplementary file 1 [file toxins-10-00246-s001.pdf]

Supplementary Materials: Intestinal Microbiota Ecological Response to Oral Administrations of Hydrogen-Rich Water and Lactulose in Female Piglets Fed a *Fusarium* Toxin-Contaminated Diet

Weijiang Zheng, Xu Ji, Qing Zhang and Wen Yao

Table S1. Effects of hydrogen-rich water and lactulose on pH values in different intestine segments of piglets fed a *Fusarium* mycotoxin-contaminated diet [1,2].

Items	NC	MC	MC+LAC	MC+HRW	SEM	*P*-value
Stomach	3.09	2.75	3.87	2.65	0.20	0.118
Duodenum	6.29	6.17	6.13	6.31	0.13	0.958
Jejunum	5.97	6.37	5.87	6.23	0.08	0.067
Ileum	6.30	6.99	6.80	6.74	0.10	0.100
Caecum	5.94	6.02	5.70	6.14	0.07	0.192
Colon	6.05	5.91	5.78	6.19	0.06	0.120
Rectum	6.45	6.80	6.94	6.70	0.07	0.070

[1] NC (negative control) = basal diet, MC = *Fusarium* mycotoxins-contaminated diet, MC+LAC = MC diet + lactulose treatment, and MC +HRW = MC diet + hydrogen-rich water treatment. [2] $n = 5$.

Table S2. Ingredient composition and nutrient contents of control and experimental diets.

Item	NC [1] diet	MC [2] diet
Ingredients, %		
Normal corn	16.75	16.75
Fusarium toxins uncontaminated corn	44.50	-
Fusarium toxins contaminated corn	-	44.50
Soybean meal	15.79	15.79
Extruded soybean	10.0	10.0
Fish meal	5.0	5.0
Wheat bran	3.0	3.0
Soybean oil	1.74	1.74
Vitamin and mineral premix[3]	1.0	1.0
Limestone powder	0.98	0.98
Calcium hydrogen phosphate	0.78	0.78
Salt	0.37	0.37
Lysine HCl (98%)	0.09	0.09
Total	100.00	100.00
Analyzed chemical composition[4]		
DM, %	88.96	88.28
CP, %	20.11	20.4
Crude ash, %	4.70	4.89
Crude fiber, %	1.71	1.96
Ether extract, %	8.04	8.65
Calculated DE,[5] kcal/kg	3,400.00	3,400.00

[1] NC, negative control (basal diet); [2] MC, mycotoxin-contaminated diet. [3] Provided, per kilogram of diet (as-fed basis) 55 mg Zn ($ZnSO_4$), 30 mg Cu ($CuSO_4$), 60 mg Mn ($MnSO_4$), 120 mg Fe ($FeSO_4$), 1 mg I (KI), 2 mg Co ($CoSO_4$), 0.3 mg Se (Na_2SeO_3), 9,000 IU vitamin A, 1,800 IU vitamin D_3, 40 IU vitamin E, 3 mg vitamin B_1, 4.5 mg vitamin B_2, 16 mg pantothenic acid, 10 mg vitamin B_6, 0.08 mg vitamin B_{12}, 28 mg niacin, 2 mg folic acid, 1.8 mg vitamin K_3, 0.2 mg biotin, 800 mg choline chloride, and 100 mg vitamin C. The premix did not contain additional Cu, Zn, antibiotics, or probiotics. [4] Unless indicated otherwise. [5] Based on a DM content of 88%.

Table S3. Primers sequences used in this study.

Target species	Primer pair	Sequence 5'-3'	Reference
Total bacteria for DGGE	U968-F-GC	CGCCCGGGGCGCGCCCCGGGCGGGGCGGGGGCA CGGGGGGAACGCGAAGAACCTTAC	
	L1401-R	CGGTGTGTACAAGACCC	[1]
Methanogenic Archaea for DGGE	519-F	CAGCCGCCGCGGTAA	
	915-R-GC	CGCCCGCCGCGCCCCGCGCCCGGCCCGCCGCCCC CGCCCCGTGCTCCCCCGCCAATTCCT	[2]
Total bacteria for qPCR	Bact1369-F	CGGTGAATACGTTCYCGG	
	Bact1492-R	GGWTACCTTGTTACGACTT	[3]
Bifidobacterium spp	Bifi-F	TCGCGTCYGGTGTGAAAG	
	Bifi-R	GGTGTTCTTCCCGATATCTACA	[4]
Lactobacilli spp.	LAC1	AGCAGTAGGGAATCTTCCA	
	Lab0677	CACCGCTACACATGGAG	[5]
Enterococcus spp.	Ent1-F	TACTGACAAACCATTCATGATG	
	Ent1-R	AACTTCGTCACCAACGCGAAC	[6]
Escherichia coli	E.coli-F	CATGCCGCGTGTATGAAGAA	
	E.coli-R	CGGGTAACGTCAATGAGCAAA	[7]
Acetogenic bacteria	FTHFS-F	TTYACWGGHGAYTTCCATGC	
	FTHFS-R	GTATTGDGTYTTRGCCATACA	[8]
Sulfate-reducing bacteria	Aps-F	TGGCAGATMATGATYMACGGG	
	Aps-R	GGGCCGTAACCGTCCTTGAA	[9]
Methanogenic *Archaea*	qmcrA-F	TTCGGTGGATCDCARAGRGC	[10]
	qmcrA-R	GBARGTCGWAWCCGTAGAATCC	

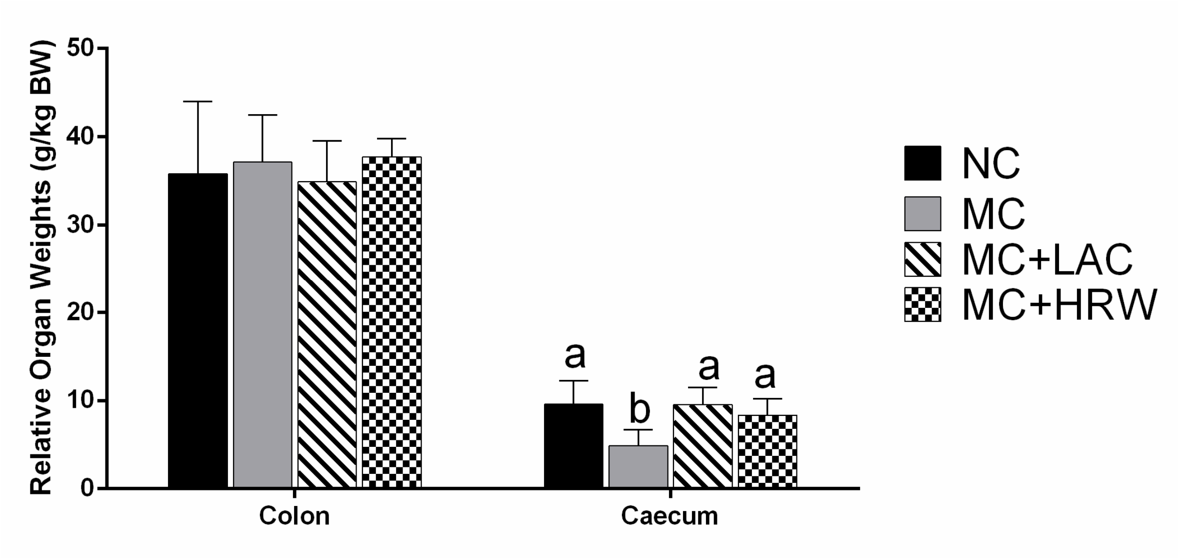

Figure S1. Effects of hydrogen-rich water and lactulose on the relative colon and caecum weights of piglets fed a *Fusarium* mycotoxin-contaminated diet. Each column represents the mean hydrogen levels with five independent replications, mean ± SD. Letters a-b above the bars indicate statistical significance ($p < 0.05$) among the four treatments. NC (negative control), basal diet; MC, *Fusarium* mycotoxins-contaminated diet; MC+LAC, MC diet + lactulose treatment; and MC +HRW, MC diet + hydrogen-rich water treatment.

Reference

1. Nubel, U.; Engelen, B.; Felske, A.; Snaidr, J.; Wieshuber, A.; Amann, R.I.; Ludwig, W.; Backhaus, H. Sequence heterogeneities of genes encoding 16s rrnas in paenibacillus polymyxa detected by temperature gradient gel electrophoresis. *J Bacteriol* **1996**, *178*, 5636-5643.
2. Cheng, Y.F.; Mao, S.Y.; Liu, J.X.; Zhu, W.Y. Molecular diversity analysis of rumen methanogenic archaea from goat in eastern china by dgge methods using different primer pairs. *Lett. Appl. Microbiol.* **2009**, *48*, 585-592.
3. Guo, X.; Xia, X.; Tang, R.; Zhou, J.; Zhao, H.; Wang, K. Development of a real-time pcr method for firmicutes and bacteroidetes in faeces and its application to quantify intestinal population of obese and lean pigs. *Lett. Appl. Microbiol.* **2008**, *47*, 367-373.
4. Walker, A.W.; Ince, J.; Duncan, S.H.; Webster, L.M.; Holtrop, G.; Ze, X.L.; Brown, D.; Stares, M.D.; Scott, P.; Bergerat, A., *et al.* Dominant and diet-responsive groups of bacteria within the human colonic microbiota. *Isme J* **2011**, *5*, 220-230.
5. Su, Y.; Yao, W.; Perez-Gutierrez, O.N.; Smidt, H.; Zhu, W.Y. 16s ribosomal rna-based methods to monitor changes in the hindgut bacterial community of piglets after oral administration of lactobacillus sobrius s1. *Anaerobe* **2008**, *14*, 78-86.
6. Ke, D.B.; Picard, F.J.; Martineau, F.; Menard, C.; Roy, P.H.; Ouellette, M.; Bergeron, M.G. Development of a pcr assay for rapid detection of enterococci. *J. Clin. Microbiol.* **1999**, *37*, 3497-3503.
7. Huijsdens, X.W.; Linskens, R.K.; Mak, M.T.; Meuwissen, S.G.M.; Vandenbroucke-Grauls, C.M.J.E.; Savelkoul, P.H.M. Quantification of bacteria adherent to gastrointestinal mucosa by real-time pcr. *J. Clin. Microbiol.* **2002**, *40*, 4423-4427.
8. Xu, K.W.; Liu, H.; Du, G.C.; Chen, J. Real-time pcr assays targeting formyltetrahydrofolate synthetase gene to enumerate acetogens in natural and engineered environments. *Anaerobe* **2009**, *15*, 204-213.
9. Deplancke, B.; Hristova, K.R.; Oakley, H.A.; McCracken, V.J.; Aminov, R.; Mackie, R.I.; Gaskins, H.R. Molecular ecological analysis of the succession and diversity of sulfate-reducing bacteria in the mouse gastrointestinal tract. *Appl. Environ. Microbiol.* **2000**, *66*, 2166-2174.

10. Denman, S.E.; Tomkins, N.; McSweeney, C.S. Quantitation and diversity analysis of ruminal methanogenic populations in response to the antimethanogenic compound bromochloromethane. *FEMS Microbiol. Ecol.* **2007**, *62*, 313-322.
